# Prioritising cardiopulmonary exercise testing for adults with cystic fibrosis: a service evaluation

**DOI:** 10.1186/s12890-026-04164-8

**Published:** 2026-02-19

**Authors:** Rachel McDowell, Chibueze Ogbonnaya, Harriet Shannon, Helen Douglas

**Affiliations:** 1 Centre for Nutrition, Exercise and Metabolism, University of Bath, Bath, UK; 2 All Wales Adult Cystic Fibrosis Centre, University Hospital Llandough, Cardiff, UK; 3 Faculty of Population Health Sciences, University College London, London, UK; 4 College of Health, Medicine and Life Sciences, Brunel University of London, London, UK

**Keywords:** Cystic fibrosis, Cardiopulmonary exercise testing, Aerobic capacity, Physiotherapy

## Abstract

**Background:**

Cystic Fibrosis is an inherited, life-limiting condition causing a range of symptoms including lowered exercise tolerance. Approximately 95% of people with cystic fibrosis in the United Kingdom are now eligible for new genetic modulator therapies. As a result, cystic fibrosis centres are treating older populations in greater numbers. Cardiopulmonary exercise testing measures aerobic capacity, however it is resource intensive. Identifying whether routinely collected clinical measures are associated with reduced aerobic capacity is needed to aid prioritisation of cardiopulmonary exercise testing.

**Methods:**

Maximal cardiopulmonary exercise testing data were collected from July 2022 to January 2024, alongside routine clinical data (spirometry, body mass index, diabetic status, Pseudomonas aeruginosa colonisation status, modulator status, age and sex). Peak oxygen uptake was analysed as a percentage predicted value (VO_2peak_pp).

**Results:**

Overall aerobic capacity at the centre was low (mean peak oxygen uptake 79.16% predicted). No relationship was identified between body mass index and aerobic capacity (β = 0.23, 95%CI -0.91, 1.37, *p* = 0.69). When adjusting for other clinical measures, having cystic fibrosis related diabetes (β=-17.56, 95%CI -27.17, -7.95, *p* < 0.001) and younger age (β = 16.62, 95%CI 4.13, 29.12, *p* = 0.01) were associated with a reduction in VO_2peak_pp.

**Conclusion:**

Annual CPET for all pwCF may not be necessary or available. This service evaluation found associations with younger age and CFRD and reduced VO_2peak_ who could be targeted for exercise testing and training intervention in the future.

**Supplementary Information:**

The online version contains supplementary material available at 10.1186/s12890-026-04164-8.

## Introduction

 The advent of cystic fibrosis transmembrane conductance regulator modulator therapies (CMT) in cystic fibrosis (CF) has resulted in an overall improvement in symptoms, and increased life expectancy [1, 2]. Multi-disciplinary teams in adult CF centres are now treating older populations and in greater numbers [2]. The CF community however are becoming more heterogeneous, meaning individualised assessment and treatment is important.

Cardiopulmonary exercise testing (CPET) is the gold standard test for measuring aerobic capacity in people with CF (pwCF), recommended to be conducted annually in the UK CF Trust Standards of Care for Good Physiotherapy Practice [3]. Only CPET accurately quantifies peak oxygen uptake (VO_2peak_) through breath-by-breath analysis, giving a highly detailed insight into the multisystem response to incremental exercise compared to other field-based exercise tests [4]. VO_2peak_ in percentage predicted (VO_2peak_pp) is a significant predictor of mortality, even after adjustment for lung function, age, body mass index, sex, lung bacterial infection and CF related diabetes (CFRD) (hazard ratio 0.964, 95%CI: 0.944–0.986) [5].

The regional All Wales Adult CF Centre, located within Cardiff and Vale University Health Board, cares for over 300 adults with CF. This number is growing by approximately 13 patients per year. In July 2022, the centre set up a CPET service for pwCF with the aim of monitoring disease and guiding exercise prescription [6]. Carrying out a CPET for each patient every year requires significant resources in terms of staff who have the time and are skilled in analysing and prescribing exercise from CPET results. Whilst guidelines state that an exercise test, ideally CPET, should be completed annually [3], this creates challenges as the adult CF patient cohort grows in number. It is also perhaps not necessary to complete a CPET annually for some patients who remain clinically well and exercising effectively regularly. The burden to patients associated with completing a CPET is significant, and the test may be unnecessary for many adults with stable and satisfactory CF health.

However, identifying those individuals who should be prioritised for CPET has not been examined before. Although spirometry, particularly forced expiratory volume in one second (FEV_1_), is a widely used outcome measure in CF care, it does not reliably identify those with reduced aerobic fitness. Up to 44% of low VO_2peak_ results seen in pwCF are attributed to generalised deconditioning rather than lung function impairment [7]. This means that relying on FEV_1_ alone to guide CPET prioritisation risks failing to identify individuals who have mild to moderate respiratory disease but are deconditioned and thus could benefit from personalised exercise prescription. Many other routine clinical measures are completed regularly at clinical reviews, such as blood samples, lung function, diabetes testing and sputum samples, however the utility of these to prioritise pwCF for CPET is unknown. Further research is needed to establish effective prioritisation strategies.

The aim of this service evaluation was to explore associations with VO_2peak_ and routinely collected clinical measures in adults (> 16 years) with CF, to enable identification of those who would gain most benefit from this form of exercise testing and prescription.

## Methods

A retrospective casenote review evaluation was undertaken. PwCF randomly undertook a CPET at a clinic review as part of their routine care between July 2022 and January 2024. People with CF were offered a CPET at clinic based on staff availability and clinic timings. All CPETs were completed on a cycle ergometer (Ergoline GmbH) and metabolic cart (Vyaire Medical) in the same designated clinical area. The modified Godfrey cycle protocol was used for all tests (continuous incremental ramp to volitional fatigue) [8]. The ramp (watt increments) was selected to aim to achieve a ten minute length test based on the software’s prediction, incorporating age, gender, height and FEV_1_, combined with the physiotherapist’s clinical assessment with the individual. Ramps used ranged between 10 and 30 watts per minute increase. Spirometry was completed before each CPET to determine FEV_1_ and predicted maximum voluntary ventilation (FEV_1_ × 35) [9]. Immediately after the test, the patient’s sense of dyspnoea and leg fatigue were self-rated on the BORG scale. The CPET data were analysed by trained physiotherapists. Anaerobic thresholds were determined using the V-slope method by identifying the inflection point on the VCO_2_ versus VO_2_ plot [10]. The cause of exercise limitation for each individual test was determined using the algorithm described in the European Respiratory Society’s statement on standardisation of cardiopulmonary exercise testing in chronic lung diseases [11], plus the clinical judgement of the physiotherapist conducting the test.

VO_2peak_pp values for each individual were calculated using normal reference values from Wasserman [12] on Sentrysuite software (Vyaire Medical). A VO_2peak_pp below 85% was deemed abnormal [11]. CPET results were included in analyses if the following conditions were met:

- were from persons with genetic confirmation of CF who were > 16 years of age and under the care of the adult service,
- met maximal criteria as per international guidelines [11],
- were the patient’s first test. If a patient had completed any subsequent tests then data from repeated tests were excluded. Longitudinal data were not available since this was a new CPET service.

Independent variables were defined (Table 1) after reviewing previously reported variables in the literature and manually extracted from electronic records and centre databases.

**Table 1:** Variable of interest and measurement instrument

Key variables of interest	Measurement instrument
Sex	Biological sex at birth
Height (cm) and weight (kg)	Stadiometer class III digital weighing scale (seca) Completed on day of CPET
CFTR modulator therapy prescription status	CMT prescriptions are collated by the pharmacy team in the All Wales Adult Cystic Fibrosis centre.
BMI	From height and weight measurements
Pancreatic status	Regular prescription of pancreatic enzyme replacement therapy
Lung transplant status	Lung transplantation occurred any time prior to the data collection point
Pseudomonas aeruginosa colonisation	Colonised: 3 positive cultures over previous 12-month period, or intermittent growth (no sputum sample within a 6 month period but grew PSa within 6 months prior to this)
	PSa free: no growth of PSa in sputum samples obtained or never grown PSa
Spirometry (FEV_1_, FVC)	Vitalograph pneumotrac or Asma handheld spirometer. Reference values from Global Lung Index 2012. Completed prior to CPET on the same day
Diabetic status	CFRD diagnosis: HbA1c > 48 or on insulin medication
	IGT: classified as IGT if the pwCF had ever previously had an impaired oral glucose tolerance test but was not currently on insulin medication or under close observation for diabetes (e.g. diet controlled)
	NGT: no history of previous oral glucose tolerance test impairment, HbA1c < 42, not on any insulin medication or under observation for diabetes (e.g. diet controlled)
C-reactive protein (mg/L)	C-reactive protein (mg/L)
White cell count (x10^9/L)	Blood results, included if taken on day of CPET
Vitamin D levels (nmol/L)	Blood results; Included if blood sample was taken on the day of the CPET.

Key: *BMI* body mass index, *CFRD* Cystic Fibrosis related diabetes, *CFTR* cystic fibrosis transmembrane conductance regulartor, *cm* centimetre, *CMT* CFTR modulator therapy, FEV_1_ = forced expiratory volume in 1 s, *FVC* forced vital capacity, *HbA1c* haemoglobin A1c, *IGT* impaired glucose tolerance, *kg* kilogram, *L* litre, *mg* miligram, *NGT* normal glucose tolerance, *PSa* pseudomonas aeruginosa;

Only data collected on the same day as the CPET were included in the service evaluation. Statistical analyses were performed using ‘IBM SPSS Statistics’ (version 29). Baseline characteristics were summarized using mean ± standard deviation (SD) for normally distributed data or median and interquartile range (IQR) for non-normally distributed data. Baseline data was summarised for the population who completed a CPET, along with the remainder of the population in the centre who did not complete a CPET to assess how representative the sample were of the rest of the Welsh population. Univariable associations (unadjusted effects) between predictor variables and VO_2peak_pp were assessed with simple linear regression. Multiple linear regression was then performed to quantify association between individual predictor variables and VO_2peak_pp after controlling for other predictors. Variables with high variance inflation factor were removed before rerunning the model [13]. Predictor variables in the regression analysis were chosen according to perceived clinical importance. Statistical model selection was also performed using the adjusted R-squared. A larger adjusted R-square implies a better fit. To prevent overfitting, the adjusted R-square penalises the R-square for the sample size and number of predictors. Variables were added to the model if they improved the adjusted R-square.

Missing data can be problematic in observational healthcare service evaluations, where data is collected for clinical reasons. We explored the missing data mechanism and found no associations between the missing values and the observed information. Therefore, we assume that missing data are missing completely at random (MCAR). This implies that there are no systematic differences between participants with observed and missing data. Pairwise analysis was used in this study when missing data was encountered. This approach for dealing with missing data gives unbiased results under the assumption of MCAR.

To further check robustness of our result to the missing data, we imputed the missing values using multiple imputation and compared regression analysis results to pairwise analysis. Multiple imputation is a method of predicting missing values while accounting for uncertainty and randomness. This involves creating multiple complete datasets, fitting the model to each dataset and combining the results. Results including the multiple imputation are presented in the supplementary information in table S2. Variables with over 50% of missing data were excluded from the study.

As the project was a service evaluation conducted by members of a clinical team, no ethics approval was required as per Health Research Authority guidelines. Cardiff and Vale University Hospital Board trust internal approval was obtained, project code Cystic Fibrosis/2023-24/01. No identifiable data were viewed by anyone outside the clinical physiotherapy team, in keeping with the principles of the Data Protection Act 1998.

## Results

**Fig. 1:**
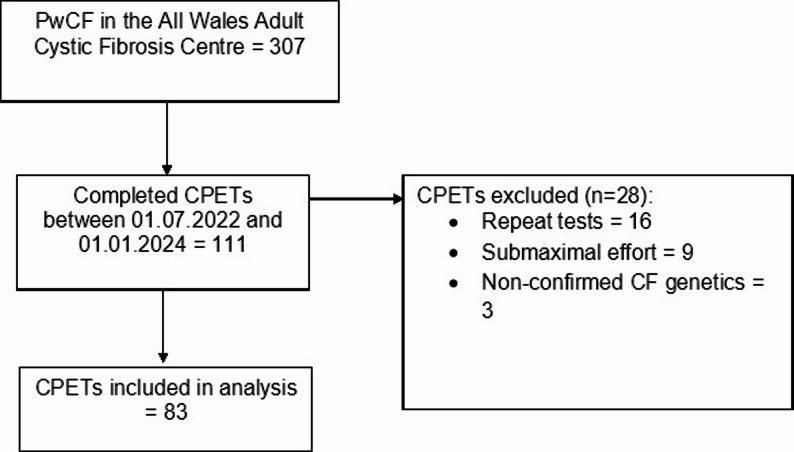
Case notes included in analyses. Key: pwCF = person with CF; CPET = cardiopulmonary exercise test

Data collection covered tests completed between 01 July 2022 and 01 January 2024 (19 months). Figure 1 details the number of CPETs completed from the total population of pwCF at the centre, and then how many of these met inclusion criteria for analyses.

### Missing data

Four (4.8%) lung function percentage predicted values were missing having been completed on handheld spirometers, and could not be retrospectively calculated. Six patients (7.2%) had missing SpO_2_% at the end of the exercise test due to artefact or signal drop out. Two patients (2.4%) were missing heart rate data due to electrocardiogram drop out during the test. Forced vital capacity was not given on eight spirometry tests (9.6%). Blood results were rarely completed on the same day as the exercise test (vitamin D, *n* = 72 missing, 86.7%, and white cell count and C-reactive protein, *n* = 64 missing, 77.1%). Due to the short half-life of vitamin D and acute changes in inflammation, blood test results were excluded from analyses.

### CPET results

The CPET results showed pwCF at the All Wales Adult Cystic Fibrosis Centre had a mean VO_2peak_pp of 79.16% (SD 17.47). VO_2peak_pp was below 85% in 60% of tests. Heart rate reached a mean of 91.7% predicted peak HR (SD 7.25). Exercise cessation was generally due to leg fatigue, with 71.1% (*n* = 59) of pwCF scoring a higher BORG for leg fatigue (18, SD 3) than dyspnoea (16, SD 5). Peak respiratory exchange ratio (RER) reached on average 1.25 (SD 0.12), and mean breathing reserve reached was 71% (SD 20.79) of calculated maximal voluntary ventilation. Among tests with an abnormal VO_2peak_pp (< 85%), 19 patients were assessed as having peripheral muscle limitation (38%), 15 were assessed as being peripherally deconditioned (30%), 7 were felt to have a cardiac limitation (15%), and 9 were ventilatory limited (18%), as defined by the ERS standardisation algorithm [11].

Baseline descriptive data (Table 2) showed pwCF who completed a CPET had a median age of 28 years (ranging from 16 to 63 years). Only 36.1% of the group were female and the disease severity was wide-ranging but on average mild (FEV_1_ 82.59pp, ranging from 28 to 117%). Table 2 also shows the demographics and clinical information for the same group when split by VO_2peak_pp threshold. Table 3 shows a comparison of the population who completed a CPET compared to the rest of the service population. The CPET sub-population differed from the wider AWACFC population having higher raw FEV_1_ and FEV_1_pp. There were no statistically significant differences in age, diabetic status, BMI, PSa colonisation status, diabetic status, pancreatic status, CMT prescription status, transplant status or sex. Participation in CPET appeared to be higher in those with a milder respiratory presentation of the disease.

**Table 2:** Demographics and clinical characteristics of pwCF who undertook CPET between July 2022 and January 2024 and were included in analysis; clinical characteristics are then described for the group when split between <85% predicted VO2peak and ≥85% predicted VO2peak on CPET results

Variable	Total (*n* = 83)	< 85% VO_2peak_ pp (*n* = 50)		≥ 85% VO_2peak_ pp (*n* = 33)
Median age, years (IQR)	28 (14)	27 (13)		29 (13)
Female sex, n (%)	30 (36.1)	16 (32)		14 (42)
Mean FEV_1_, L (SD)	3.09 (0.93)	3.07 (1.01)		3.12 (0.81)
Mean FEV_1_pp, % (SD)	82.59 (19.9)	81.8 (22.08)		83.69 (16.66)
Mean FVC, L (SD)	4.15 (0.99)	4.09 (1.04)		4.23 (0.91)
Mean BMI (SD)	23.92 (2.38)	23.84 (3.64)		24.05 (2.7)
Previous lung transplant, n (%)	4 (4.8)	4 (8)		0 (0)
Pancreatic insufficient, n (%)	70 (90.4)	46 (92)		29 (87.9)
PSa colonised, n (%)	35 (42.4)	22 (44)		13 (39.39)
Diabetic status				
CFRD, n (%)	18 (21.7)	17 (34)		1 (3)
IGTT, n (%)	31 (37.3)	15 (30)		16 (48.5)
NGT, n (%)	34 (41)	18 (36)		16 (48.5)
CMT status				
CMT prescribed, n (%)	72 (86.7)	44 (88)		29 (87.9)
No CMT, n (%)	11 (13.3)	6 (12)		4 (12.1)

Key: *BMI* body mass index, measured as weight (kg) / [height (m)]2, *CFRD* CF related diabetes, *CI* confidence interval, *FEV1* forced expiratory volume in 1 second (percentage predicted values calculated using global lung index), *CMT* CFTR modulator therapy, *IGT* impaired glucose tolerance, *NGT* normal glucose tolerance, *pp* percentage predicted, *PSa* pseudomonas aeruginosa. Data given as mean unless specified median (as data skewed)

**Table 3:** Comparison of CPET subpopulation to wider AWACFC population

	CPET population (*n* = 83)	Total AWACFC population (*n* = 307)	Mean difference (95% CI)	*p* value
Sex (= female, %)	30 (36.1)	139 (41.3)		0.934
Age (years)	28.00	32.00		0.071
FEV_1_ (L)	**3.09**	**2.81**	**0.28 (0.08**,** 0.48)**	0.008*
FEV_1_ pp	**82.59**	**75.66**	**6.93 (2.48**,** 11.39)**	0.003*
Previous lung transplant (%)	4 (4.8)	19 (5.1)		1.000
CMT status (%)				
CMT prescribed	72 (86.7)	275 (76.6)		0.063
No CMT	11 (13.3)	84 (23.4)		
Pancreatic insufficient	75 (90.4)	222 (77.4)		0.079
Diabetic status (%)				
CFRD	18 (21.7)	106 (28.3)		0.085
Previous IGT	31 (37.3)	101 (26.9)		
NGT	34 (41)	168 (44.8)		
PS*a* colonisation (%)	35 (42.2)	131 (43)		0.864
BMI	23.9	24.5	-0.58 (-1.29, 0.14)	0.114

Key: *BMI* body mass index, *CFRD* cystic fibrosis related diabetes, *CMT* CFTR modulator therapy, *FEV*_*1*_ forced expiratory volume in 1 s, *FVC* forced vital capacity, *IGTT* previously impaired glucose tolerance test, *IQR* interquartile range, *L* litres, *NGT* normal glucose tolerance, *pp* percentage predicted, *PSa* pseudomonas aeruginosa, *SD* standard deviation, *VO*_*2peak*_ peak oxygen uptake

* = p<0.05

### Predictor variables

No significant relationship was identified between BMI and VO_2peak_pp (Table 4). As BMI increased by one point, VO_2peak_pp changed by -0.96 to 1.36 (0 = 0.729) showing no clear positive or negative relationship. Simple linear regression showed no relationship between sex, age, PS*a* colonisation, being prescribed CMT or FEV_1_pp and VO_2peak_pp (Table 4).

**Table 4:** Regression coefficients for predictor variables on VO2peakpp in PwCF at the all Wales adult cystic fibrosis centre

	Univariable regression coefficients		Multivariable linear regression coefficients	
Variable	VO_2peak_pp β (95% CI)	*p*	VO_2peak_pp β (95% CI)	*p*
Age (log)	5.4 (-6.39, 17.19)	0.365	16.62 (4.13, 29.12)	0.01
FEV_1_% predicted	0.14 (-0.06, 0.33)	0.177	0.14 (-0.06, 0.34)	0.166
Sex = Male	-5.76 (-13.65, 2.13)	0.15	-7.55 (-15.46, 0.37)	0.061
BMI	0.49 (-0.68, 1.66)	0.406	0.23 (-0.91, 1.37)	0.689
PSa status = colonised	-4.66 (-12.36, 3.05)	0.232	-2.9 (-10.57, 4.76)	0.452
CMT status = prescribed	-0.57 (-11.89, 10.75)	0.92	-0.53 (-11.53, 10.47)	0.924
Diabetic status= CFRD	-0.57 (-25.35, -8.26)	< 0.001	-17.56 (-27.17, -7.95)	< 0.001

Key: *BMI* body mass index, *CFRD* Cystic Fibrosis related diabetes, *CI* confidence intervals, *ETI* elexacaftor/tezacaftor/ivacaftor, *FEV*_*1*_ forced expiratory volume in 1 s, *CMT* CFTR modulator therapy, *PSa* pseudomonas aeruginosa, *pwCF* person with cystic fibrosis, *VO*_*2peak*_*pp* peak oxygen uptake, *pp* per centage predicted, *β* coefficient, *n* sample size

For diabetes status, the IGTT group were combined with the non-diabetic group due to a high correlation between the two (*r*=-0.677). Having a diagnosis of CFRD was associated with a statistically significantly lower VO_2peak_pp by between 27.17 and 7.95%, compared to no CFRD after adjusting for other model predictors (*p* < 0.001) (Table 4). Age also had a statistically significant association: each unit increase in (log-transformed) age was associated with a 16.624 (95% CI: [4.13, 29.12], *p* = 0.01) increase in VO_2peak_pp after adjusting for the other model predictors. Each 1% increase in age was associated with VO_2peak_pp increasing by 0.17, with the true increase in VO_2peak_pp lying between 0.04 and 0.29% per 1% increase in age. All other predictors were non-statistically significant.

### Sensitivity analysis

When predictor variables in the multivariable linear regression model were chosen using adjusted R-squared, the results remained consistent with a significant association between log of age, diabetes status and VO_2peak_pp (Table S1). This model had a slightly higher adjusted R-squared (22.8%) compared to the model that included all the predictors (20.7%).

To assess the robustness of our results to the missing data in this study, we performed multiple imputation with five imputed datasets and compared to the pairwise analysis results. The multivariable linear regression results from multiple imputation were similar to those from pairwise deletion, therefore confirming the robustness of our results (Table S2).

## Discussion

European standards for future care stress the importance of treating pwCF as individuals [14], however previous guidance has advocated for an annual “test all” approach to assessment of exercise capacity [3]. This study aimed to identify routinely collected clinical factors associated with reduced VO_2peak_pp in pwCF to guide prioritisation of CPET to those who would most benefit from formal exercise prescription. It is the first known study to explore these associations in a cohort primarily receiving CMT. Despite medical advancements, 60% (*n* = 50) of pwCF at the centre demonstrated a reduced VO_2peak_pp, reinforcing the need for continued monitoring and intervention to mitigate long-term health risks [5].

In this cohort of adults with cystic fibrosis, the cause of exercise limitation was frequently attributable to non-pulmonary factors, with CPET identifying physical deconditioning in approximately 30% of participants and peripheral muscle limitation in 38%. Although these mechanisms may coexist, they reflect distinct pathophysiological processes: physical deconditioning is primarily secondary to reduced habitual physical activity and is potentially reversible with exercise prescription [15], whereas peripheral muscle limitation reflects intrinsic skeletal muscle dysfunction related to cystic fibrosis, including impaired oxidative capacity, altered muscle fibre composition, systemic inflammation, and nutritional compromise [16]. The relatively high prevalence of peripheral muscle limitation observed in this study highlights that exercise intolerance in cystic fibrosis cannot be solely attributed to inactivity and underscores the value of CPET in delineating underlying mechanisms to inform targeted exercise and rehabilitation strategies [17].

Similar levels of exercise intolerance have been reported internationally [18], although higher VO_2peak_ values have been observed in studies using treadmill CPET [7] or supramaximal verification protocols [19, 20]. Differences in aerobic fitness across studies may be influenced by variations in CPET protocols, health behaviours, and sample selection. Recent longitudinal evidence also suggests that expected developmental gains in aerobic fitness may not be fully realised in pwCF. Gaupmann et al. (2025) reported that incremental increases in VO_2peak_ among children and adolescents aged 12–20 years were markedly lower than those predicted by reference values, indicating a blunted trajectory of aerobic capacity despite advances in CF care. This aligns with our findings of widespread reduced VO_2peak_pp in adulthood and reinforces the need for continued monitoring across the lifespan [21]. Additionally, the emerging increased risk of metabolic and cardiovascular diseases in pwCF makes our findings concerning [22].

Contrary to prior literature linking BMI to VO_2peak_pp, this evaluation found no significant association, suggesting other factors play a more significant role potentially due to improved nutritional status with CMT [2, 23]. BMI may now be too crude a measure, as body composition – specifically fat-free mass – has shown stronger correlations with muscle strength [24] and exercise tolerance [25, 26] in paediatric populations. Whilst preliminary service evaluations show significant changes in body composition in pwCF on initiating CMT [27], the long-term effects are unknown. As clinician attitudes to nutrition change in response to changes in CF phenotype [28], tackling overweight and obesity through healthy living recommendations may alter prioritisation for CPET in the All Wales Adult Cystic Fibrosis Centre for which body composition knowledge will be key.

Only a small number of the population who completed a CPET were female (36%). Despite there being a smaller proportion of females with CF in Wales than males, it appears there is a still smaller proportion of females who undertook a CPET. It is possible that females are less likely to be offered a test due to pregnancy, may be more likely to be exacerbating [29], or are more likely to be colonised with bacterial infections which contraindicate use of gas exchange measurement equipment on CPET [30]. An analysis in a different CF centre in the United Kingdom found no significant associations between sex and uptake of CPET (*p* = 0.34), indicating that elsewhere females are not more likely to refuse a CPET when offered [31]. However, this could still have been a possibility at our service, but records of those who declined testing were not analysed as part of this service evaluation.

This evaluation found CFRD were significantly associated with reduced VO_2peak_pp in pwCF, similar to findings by Causer et al. [32] though their findings lost significance when lung function was included as a covariate in analyses. The lung function of the different diabetic status groups differed significantly in Causer et al. (2020) compared to our service evaluation which may explain the difference in the findings. The diabetic group at our centre may have a better-preserved respiratory function compared to the diabetic group in Causer et al. (2020) due to having had access to CMT since 2021, however they remained statistically significantly limited in their aerobic capacity compared to non-diabetic or impaired glucose tolerance test pwCF. This service evaluation also included pseudomonas aeruginosa colonisation status as a predictor, which differed from previous studies.

CFRD may impact aerobic fitness through multiple mechanisms. Diabetic pathophysiology accelerates known microvascular dysfunction associated with CF [33]. It is therefore attractive to hypothesise that more advanced microvascular dysfunction would lower VO_2peak_ due to decreased oxygen uptake within skeletal muscle cells from muscle capillaries. Behaviour change may be one driver of the results seen in the service evaluation; it is possible that the increased treatment demands of having diabetes additionally to CF may mean physical activity and exercise become more difficult to fit into a daily routine [34], therefore leading to a lower VO_2peak_pp than that of someone without CFRD. Indeed, when physical activity is controlled for (using step counts from pedometers), no differences in VO_2peak_ have been found [35].

Older age was associated with a small increase in VO_2peak_pp differing from prior findings where age had no significant effect [7, 32]. Given the typical trajectory of progressive physiological decline in CF, this association was unexpected and should be interpreted cautiously. Several explanations may account for this finding. Survival bias is a plausible confounder, as fitter individuals are more likely to reach older age [5]. Given only a small number of pwCF were aged older than 40 years in our study (15.7%), further research with larger samples is needed. Consequently, our findings should be viewed as preliminary, and further studies with larger and more age-diverse samples are needed to clarify this association.

**Fig. 2:**
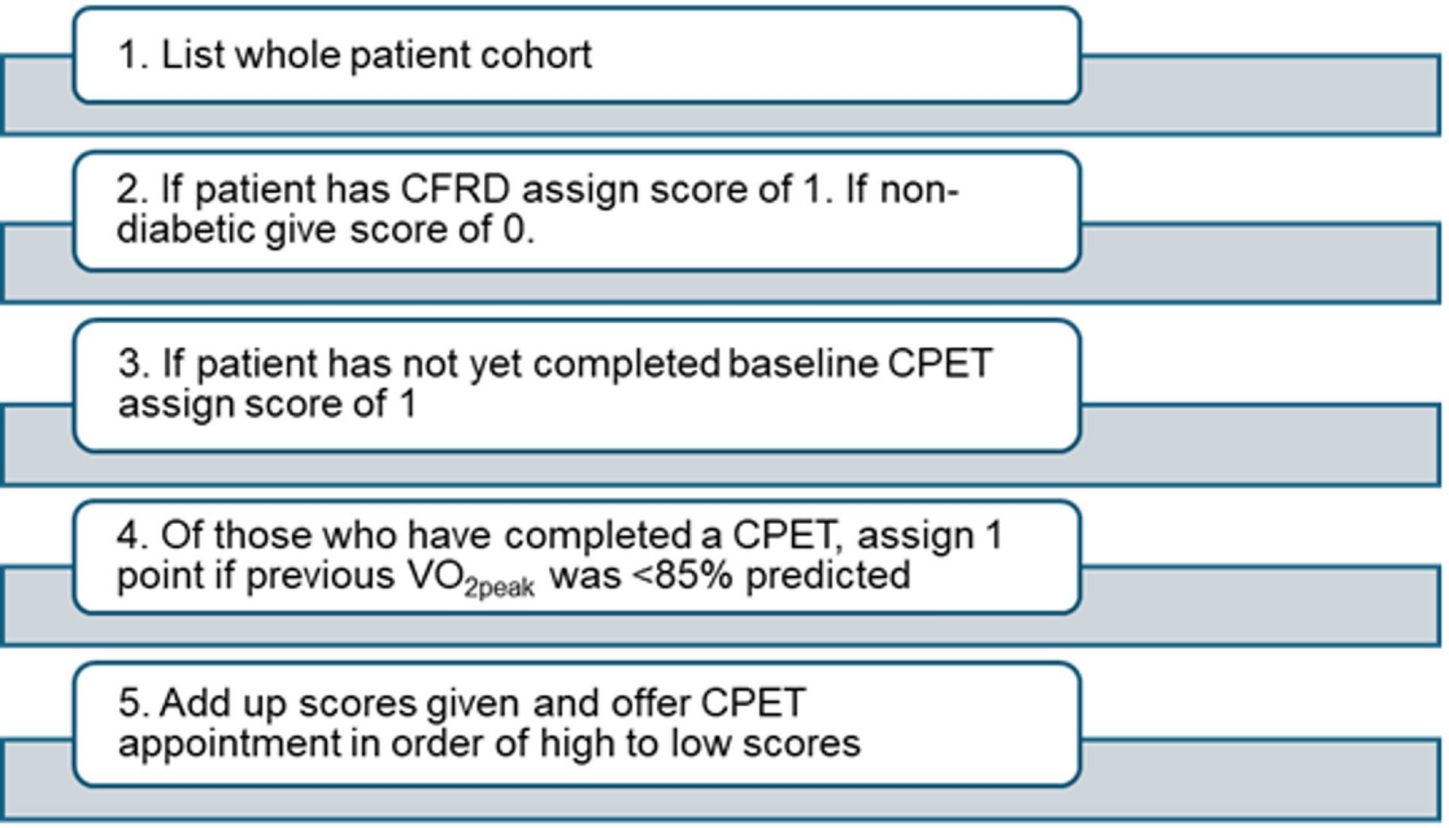
Suggested CPET prioritisation tool. Key: CPET = cardiopulmonary exercise testing; CFRD = Cystic Fibrosis related diabetes

The significant impact of CFRD status on VO_2peak_pp in the All Wales Adult Cystic Fibrosis Centre population suggests CPET could be prioritised for those with diabetes to optimise long-term health outcomes. From the results of the service evaluation we propose an algorithm (Fig. 2) to use in clinical practice to prioritise selection for CPET from clinics at the All Wales Adult Cystic Fibrosis Centre. Future service evaluations should look to re-evaluate this algorithm, regularly updating as the paradigms of CF care changes.

### Strengths and limitations

The inclusion of only maximal CPETs was a strength of the service evaluation by reducing the impact of low motivation on results [36]. Furthermore, only variables recorded on the day of CPET were included, minimising inaccuracies that could arise from using non-concurrent predictor data. However, several limitations should be acknowledged. The service evaluation was conducted at a single centre using retrospectively collected results. The use of Wasserman’s normal reference values for VO₂_peak_ are widely used worldwide however, alternative normal reference values specifically recommended for cycle ergometry in pwCF [37] could be considered in future studies. Additionally, while VO₂_peak_pp was selected as the primary outcome due to its clinical relevance, other CPET-derived measures, such as peak work rate [38], could provide complementary insights. The evaluation also lacked routinely collected physical activity data, despite its known association with aerobic capacity in pwCF. Shelley et al. (2018) highlighted the potential benefit of incorporating wearable activity trackers or validated questionnaires in future assessments [39]. Another inherent limitation of using clinical data for evaluation was the presence of missing data, despite handling of this in the sensitivity analyses completed. Finally, a small number of repeat CPETs were excluded, though ongoing CPET assessments at the All Wales Adult Cystic Fibrosis Centre will allow future service evaluations to incorporate longitudinal data.

Future studies should investigate mechanisms underlying reduced VO_2peak_pp in pwCF, particularly the effects of age and CFRD. Longitudinal research could assess how changes in predictor variables influence VO_2peak_pp, while additional studies should explore factors such as body composition, physical activity, inflammation, and vitamin D, all of which were unable to be included in the service evaluation. The long-term impact of CMT remains unclear, highlighting the need to refine exercise testing and prescription strategies in the growing population with CF.

## Conclusions

CF care must evolve with increasing patient numbers and changing disease phenotypes. Annual CPET for all pwCF may not be necessary: the CF community may become ever more heterogeneous as a clinical population meaning individualised assessment and treatment of exercise capacity is needed. Resources should be prioritised to those who would likely benefit the most from an exercise assessment and prescription. This service evaluation is the first to examine routine clinical measures associated with VO_2peak_pp in a predominantly CMT-treated cohort. Unlike previous studies, which linked reduced VO_2peak_pp to BMI, this service evaluation found an association with CFRD, and more cautiously with younger age. The mechanisms behind this, along with CF’s shifting nutritional profile, require further investigation. Despite CMT, many pwCF still exhibit low VO_2peak_pp, underscoring the need to offer exercise training input, especially if deconditioned. For those with a normal CPET, we could assume that we can leave longer between testing to help with prioritisation of long-term CF health management in a fiscally constrained healthcare system.

## Supplementary Information

Supplementary Material 1.

## Data Availability

The datasets used and/or analysed during the current study are available from the corresponding author on reasonable request.

## Abbreviations

BMI: Body mass index
CF: Cystic Fibrosis
CFRD: Cystic fibrosis related diabetes
CI: Confidence intervals
CPET: Cardiopulmonary exercise testing
FEV_1_: Forced expiratory volume in one second
IGTT: Previously impaired glucose tolerance test
NGT: Normal glucose tolerance
Pp: Percentage predicted
PSa: Pseudomonas aeruginosa
pwCF: People with cystic fibrosis
RER: Respiratory exchange ratio
SD: Standard deviation
VO_2peak_pp: Peak oxygen uptake as a percentage predicted value
