# Supplementary material for: Prioritising cardiopulmonary exercise testing for adults with cystic fibrosis: a service evaluation
Source: BMC Pulm Med. 2026 Feb 19;26:134. doi: 10.1186/s12890-026-04164-8 (PMC13032233; doi:10.1186/s12890-026-04164-8)
Supplement: Supplementary file 1 — Supplementary Material 1. [file 12890_2026_4164_MOESM1_ESM.docx]

| Table S1: Reduced Multivariable linear regression with variable selection using adjusted R-square | | | |
| --- | --- | --- | --- |
| Variable | **β** | **95% CI** | **P-value** |
| Age(log) | 15.89 | (4.22, 27.56) | 0.008 |
| FEV1 | 0.14 | (-0.05, 0.33) | 0.148 |
| Sex=Male | -6.13 | (-13.53, 1.26) | 0.103 |
| PSa Status = colonised | -3.61 | (-10.96, 3.74) | 0.331 |
| Diabetes status = CFRD | -19.27 | (-28.04, -10.51) | <0.001 |

*Key: CI = confidence intervals; CFRD = cystic fibrosis related diabetes, FEV1 = forced expiratory volume in one second; PSa = pseudomonas aeruginosa colonisation; β = coefficient*

| Table S2: Results from Multivariable linear regression with multiple imputation | | | |
| --- | --- | --- | --- |
| Variable | **β** | **95% CI** | **P-value** |
| Age(log) | 15.06 | (3.39, 26.74) | 0.011 |
| FEV1 | 0.13 | (-0.06, 0.32) | 0.190 |
| Sex=Male | -6.52 | (-13.84, 0.80) | 0.081 |
| BMI | 0.26 | (-0.85, 1.37) | 0.644 |
| PSa Status = colonised | -3.40 | (-10.72, 3.92) | 0.362 |
| CMT Status | -0.68 | (-10.45, 9.09) | 0.891 |
| Diabetes status = CFRD | -19.44 | (-28.20, -10.68) | <0.001 |

*Key: CI = confidence intervals; CFRD = cystic fibrosis related diabetes, FEV1 = forced expiratory volume in one second; PSa = pseudomonas aeruginosa colonisation; β = coefficient*
